# Current perspectives on invasive nontyphoidal Salmonella disease

**DOI:** 10.1097/QCO.0000000000000398

**Published:** 2017-10-01

**Authors:** Andrea H. Haselbeck, Ursula Panzner, Justin Im, Stephen Baker, Christian G. Meyer, Florian Marks

**Affiliations:** a International Vaccine Institute, Epidemiology Unit, Seoul, Republic of Korea; b Hospital for Tropical Diseases,Wellcome Trust Major Overseas Programme, Oxford University Clinical Research Unit, Ho Chi Minh City,Vietnam; c Department of Medicine, University of Cambridge, Cambridge,United Kingdom; d Institute of Tropical Medicine, Eberhard-Karls University T€ubingen, T€ubingen, Germany and; e Duy Tan University, Da Nang, Vietnam

**Keywords:** epidemiology, invasive nontyphoidal Salmonella disease, invasive, nontyphoidal Salmonella, vaccination

## Abstract

**Purpose of review:**

We searched PubMed for scientific literature published in the past 2 years for relevant information regarding the burden of invasive nontyphoidal Salmonella disease and host factors associated with nontyphoidal Salmonella infection and discuss current knowledge on vaccine development. The following search terms were used: Salmonella, non typhoidal/nontyphoidal, NTS, disease, bloodstream infection, invasive, sepsis/septicaemia/septicemia, bacteraemia/bacteremia, gastroenteritis, incidence, prevalence, morbidity, mortality, case fatality, host/risk factor, vaccination, and prevention/control.

**Recent findings:**

Estimates of the global invasive nontyphoidal Salmonella disease burden have been recently updated; additional data from Africa, Asia, and Latin America are now available. New data bridge various knowledge gaps, particularly with respect to host risk factors and the geographical distribution of iNTS serovars. It has also been observed that Salmonella Typhimurium sequence type 313 is emergent in several African countries. Available data suggest that genetic variation in the sequence type 313 strain has led to increased pathogenicity and human host adaptation. A bivalent efficacious vaccine, targeting Salmonella serovars Typhimurium and Enteritidis, would significantly lower the disease burden in high-risk populations.

**Summary:**

The mobilization of surveillance networks, especially in Asia and Latin America, may provide missing data regarding the invasive nontyphoidal Salmonella disease burden and their corresponding antimicrobial susceptibility profiles. Efforts and resources should be directed toward invasive nontyphoidal Salmonella disease vaccine development.

## INTRODUCTION

Salmonella senterica serovar Typhi (S. Typhi) and the various pathovars ofs S. Paratyphi are commonly re-ferred to as typhoidal Salmonella serovars. These agents are restricted to human hosts. Salmonella serovars that fall outside of this group are typically referred to as the nontyphoidal Salmonella (NTS) serovars and are considered to have the potential to interact with human and nonhuman hosts [1■■]. Poor access to improved water supplies and adequate sanitation facilities, combined with growing urbanization, favor the transmission of NTS serovars through food or water sources and contact with animals [2■■]. In addition to animal reservoirs, humans may be a growing substantial secondary pathogen reservoir [3]. Typical NTS disease in immunocompetent hosts manifests as a mild, self-limiting gastroenteritis. In contrast, invasive nontyphoidal Salmonella (iNTS) disease commonly presents as a febrile bacteremia, which can be fatal if left untreated. Invasive NTS disease is associated with the extremes of age, malnutrition, clinical malaria, and HIV infections, especially in Africa [4■,5■■–7■■]. In this study, we review available literature published from 2015 to present on the global burden of iNTS disease, host risk factors, and the implications of these data for vaccination.

### KEY POINTS

• Global estimates suggest 3.4 million iNTS illnesses and
- 618 316 iNTS diseaserelated deaths per year.
• The most common iNTS serovars are S. Enteritidis,
- *S. Dublin, S. Typhimurium*; of particular concern is the
- *sS. Typhimurium* ST313 variant.
• Bivalent *S. Enteritidis* and *S. Typhimurium* vaccines may decrease the global disease burden dramatically.

## GLOBAL DISEASE BURDEN

A recent review on the global distribution of iNTS has indicated a low number of reported cases in South and Southeast Asia [8■]. The overwhelming majority of the estimated 3.4 million annual iNTS infections and 618 316 iNTS-related deaths occur in Africa [4■,8■,^9^]. Salmonella enterica serovars Enteritidis, Dublin, and Typhimurium are the most common serovars associated with iNTS disease [4■,5■■]. Of specific concern is Typhimurium sequence type 313 (ST313), which is more frequently associated with bacteremia than gastrointestinal infections [10,11], and is commonly multidrug resistant (MDR) [2■■,^12^]. Additional data suggest that S. Typhimurium ST313 may have a greater propensity for transmission between humans, and an animal reservoir has not, as yet, been well defined. Keddy et al. [13] recently found a significant association of an MDR phenotype in ST313 [odds ratio (OR) 6.6; 95% confidence interval (95%CI) 2.5 – 17.2] in comparison to S. Typhimurium sequence type 19 (ST19). ST19 arises mostly in Europe and Northern America, whereas ST313 isolates are more commonly found in Africa [14■■].

A review published in 2017 assessed the occurrence of iNTS disease across Africa [5&&]. The authors described disease incidence estimates that ranged from 1.4 per 100 000 population/year (all ages, South Africa, 2003 – 2004) to 2520 per 100 000 population/ year (children <5 years old, Ghana, 2007 – 2009) [5■■], with highest incidences in those infected with HIV, in patients with sickle cell disease, in young children, and in those residing in rural settings [5]. The prevalence of NTS-related community-acquired bacteremia ranged from 8% in Nigeria and South Africa to 45% in the Central African Republic [5■■], with an overall case fatality rate of 20.6% (548 deaths/2656 iNTS disease cases) [5■■].

The emergence of iNTS organisms in Africa exhibiting resistance to various commonly used antimicrobials, including chloramphenicol, ampicillin, and co-trimoxazole, has been reported [2■■,4■,^15^,^16^]. These ‘baseline’ antimicrobial resistance profiles have been followed by the advent of resistance against third-generation cephalosporins; iNTS isolates with resistance to ceftriaxone has now been reported in the Democratic Republic of the Congo (DRC) [17,18], Kenya [19,20], Malawi [15,21] and South Africa [2■■].

The Typhoid Fever Surveillance in Africa Program (TSAP), a population-based surveillance, con-ducted at 13 sentinel sites in 10 countries (Burkina Faso, Ethiopia, Ghana, Guinea-Bissau, Kenya, Madagascar, Senegal, South Africa, Sudan, and Tanzania) during 2010 – 2014, revealed an overall iNTS disease prevalence of 17% (94/568) among those with bacteremia [22]. The serovars S. Typhimurium (40%, 38 out of 94 NTS positive cases), S. Enteritidis (12%, 11 out of 94 NTS positive cases) and S. Dublin (11%, 10/94 NTS positive cases) were the most prevalent [22 – 24], which is largely concordant with findings reported by Crump and Heyderman [4■] and Uche et al. [5■■]. In TSAP, the adjusted incidences of iNTS disease were highest among children aged more than 1 year, ranging from 291 (95%CI 176 – 482) per 100 000 person-years-of-observations (PYO) (Guinea-Bissau) to 1733 (95%CI 1373 – 2188) per 100 000 PYO (Ghana) [22]. The iNTS disease incidences among children aged 2 to 4 years ranged from 49 (95%CI 7 – 348) per 100 000 PYO in Kenya to 1908 (95%CI 1469 – 2479) per 100 000 PYO in Ghana [22]. In addition, several independent reports on blood-culture-based surveillance data have shown that iNTS disease is present in other locations in Africa such as the DRC [18], the Gambia [25], and Ghana [26,27]. Data from different sites in Kenya found an incidence of 4134 per 100 000 person-years [20] and 174 per 100 000 person-years [28] in infants. Children under 5 years of age had an overall incidence of 36.6 per 100 000 person-years [28,29]; incidences among children less than 5 years of age differed considerably by setting (rural setting: 3914 per 100 000 person-years, urban setting: 997.9 per 100 000 person-years) [30]. The presence of iNTS disease has also been reported from Mali [31], Mozambique where two studies were conducted [predominantly ST313 isolates [32]; infant incidence: 217.7 per 100 000 child-years [33■]], and South Africa [34■■].

In contrast to Africa, the epidemiology of iNTS disease and corresponding antimicrobial susceptibility patterns are poorly described in Asia and South America, suggesting either a lower disease burden or a lack of epidemiological reporting. A multicenter, hospital-based study investigating community-acquired bacteremia in Indonesia, Thailand, and Vietnam from 2013 to 2015 identified an overall NTS-associated bacterial positivity rates of 27.5% (11/40 bacteremia cases) in children and 11.7% (7/60 bacteremia cases) in adults [35]. Limited iNTS prevalence (20/12 940 bacteremia patients) and a 25% case fatality were reported among bacteremic patients hospitalized from 2009 to 2013 in Bangladesh [36]. A longitudinal study of community-acquired bacteremia in hospitalized children con-ducted in Malaysia from 2001 to 2011 found an iNTS prevalence of 16.2% (36/222), with most NTS isolated from infants below 1 year of age [37]. A surveillance study from Colombia investigated a sample of 4010 S. Enterica isolates collected from blood and feces samples and found that 32.5% were S. Typhimurium, 28.2% were S. Enteritidis, and 2.9% were S. Dublin cases over a 6-year period [38]. These numbers are considerably lower than those reported from Asia and sub-Saharan Africa. Notably, S. Typhimurium ST313 variants have been isolated from humans and poultry in Brazil [39]. On the basis of the investigations of Almeida et al. [39], the organ-isms identified appear genetically distinct from the ST313 variants isolated in sub-Saharan Africa.

## HOST-ASSOCIATED FACTORS

Common factors contributing to iNTS disease include extremes in age, the occurrence of immunosuppressive conditions, and other underlying comorbidities (e.g., diabetes, cancer, and cardiovascular diseases) [40]. In addition, climatic conditions such as increased rainfall or drought that can result in food scarcity, leading to malnutrition and in-creased transmission of malaria parasites are factors that may favor the transmission of NTS organisms [7■■]. Particularly in Africa, the association of iNTS disease with malnutrition (OR 1.44 – 2.42) and sickle cell disease (OR 35.6) has been described predominantly in children, whereas Plasmodium falciparum malaria (OR 1.5 – 4.1), anemia, and HIV infection (OR 3.2 – 48.2) are risk factors that are not generally associated with age [2■■,4■,5■■,7■■,^24^,^29^,41■■]. Adjusted odds ratios of 4.0 and 5.0 were calculated for the association of iNTS disease with moderate and severe anemia, respectively [33■]. Keddy et al. [34■■] found a significant association between an increased usage of antiretroviral therapy and a de-crease in incident iNTS disease infections (P < 0.001) in a South African province. Similar observations were made by Lan et al. [42] in Vietnam. ST313, the most common S. Typhimurium variant associated with iNTS disease, was initially identified in HIV-infected patients [2■■]. In scomparison to other S. Typhimurium types (e.g., ST19), the genomically degraded ST313 may cause systemic infections and induce a lower inflammatory reaction in the intestine, exerted by evasion mechanisms from the immune response [43,44]. The genomic degradation includes the downregulation of gene expression involved in active cell invasion through effector proteins [43,44]. The reduced activation of macrophages is assumed to be caused by lower flagellin expression [43,44]. The survival time and replication rate were found to be more efficient in the investigated ST313 isolates compared with ST19 [43,44]. Therefore, the ST313 phenotype appears to become closer to that of typhoidal Salmonella, suggesting analogical adaptation toward a more invasive phenotype in humans [1■■].

In addition, an MDR phenotype may allow for rapid ST313 dissemination throughout susceptible populations [2■■]. Advanced HIV disease leads to a reduced immune response in the gastrointestinal mucosa and poses a higher invasion risk of iNTS [7■■]. Changes in the gastrointestinal microbiota, induced by the intake of acid blockers, gastric surgery, and antimicrobial pretreatment, are also suggested to favor iNTS disease [7■■,^45^,^46^]. Martz et al. [46] found stabilizing effects on the gastro-intestinal microbiome associated with the ingestion of probiotics in mice, which may improve the functionality of the intestinal barrier.

## VACCINE DEVELOPMENT

Effective vaccines preventing iNTS disease are likely to differ inherently from those protecting against S. Typhi infections. Studies from Africa have shown that naturally acquired antibodies against NTS correspond with a reduced risk of iNTS disease [47,48]. Several vaccine candidates targeting S. Typhimurium and S. Dublin sare currently under development, some of which may provide protection against both serovars. The current status of iNTS vaccine considerations has been described in a recent review [49■■]: several potential iNTS vaccines are under development, including live-attenuated, subunit-based, and recombinant antigen-based substances. Both humoral and cellular immunities are likely required to achieve full protection against iNTS disease. Live-attenuated vaccines provide both types of immune response; however, they may pose a risk for immunocompromised individuals [41■■]. Inactivated iNTS vaccines may induce humoral immunity only and suppress NTS during the acute phase of infection, but are likely not to achieve systematic clearance in infected individuals [41■■]. The lack of disease burden data from Asia and South America, coupled with the enormous number of NTS serovars, and the role of alternate prevention measures (i.e., access to improved water and sanitation and food safety) have contributed to a delay and a lack of investment in the development of iNTS disease vaccines.

Previously, when animals were considered to be the only reservoir of NTS organisms, the implementation of hygiene and safety measures along a regulated and appropriate food chain was thought to be sufficient for the reduction of iNTS transmission. However, with the speculation that humans may be a growing alternative reservoir for ST313 [50], the development and deployment of iNTS disease vaccines appear to be a more viable solution. However, iNTS disease vaccines would not only require considerable funding to progress existing vaccine candidates, but also will require parallel vaccine deployment strategies to identify appropriate target age groups, schedules, formulations, and potential vaccine adjuvants.

An iNTS disease vaccine would need to be ad-ministered in infants to ensure protection before the peak occurrence of disease. This strategy, however, poses the challenge of combining iNTS and S. typhi vaccines, as the peak disease incidence for typhoid (5 – 8 years of age) is later than that for iNTS disease [51], except in highly endemic areas. A potential byproduct after the widespread use of a bivalent iNTS vaccine conferring protection against S. Typhimurium and S. Enteritidis would be serovar replacement by other Salmonella variants, such as S. Dublin. Such a serotype replacement was observed following the MenAfriVac campaign, when large populations in the African meningitis belt were vaccinated against Neisseria meningitidis serotype A and other serotypes subsequently emerged [52 – 54]. Another consideration would be a combined iNTS disease/malaria vaccine; this approach may be particularly prudent, given that malaria is associated with the severity of iNTS disease. Such a vaccine would then be tailor-made for sub-Saharan Africa, but may be less applicable for low and nonendemic malaria regions (i.e., Brazil) [39]. This would potentially suggest the need to develop an independent, nonmalaria-combined vaccine that is applicable to all iNTS endemic regions.

## conclusions

iNTS is a major public health issue in sub-Saharan Africa. ST313 appears to be better adapted to humans than other S. Typhimurium, is associated with an increased disease severity, and has acquired an MDR phenotype. Observations of the Brazilian ST313 lead to some insights on this serotype that are also relevant for Africa. First, ST313 has the ability to arise in new locations independently and does not appear to be confined to sub-Saharan Africa. The fact that some Brazilian ST313 isolates exhibit different antimicrobial susceptibility profiles in comparison to African variants suggests that iNTS disease has the potential to evolve de novo outside of Africa, which may result in new and unlinked epidemics. Improvement in water and sanitation, a reduction in malaria incidences and malnutrition and improved management of HIV infections should additionally prevent iNTS disease from becoming an even bigger global health threat. However, the rapid emergence of ST313, the possible de-novo occurrence and spread of the future MDR and pathogenic variants place iNTS disease increasingly on the vaccine development agenda. Now is also a prime time to invest in enhanced iNTS disease surveillance. This enhanced surveillance is particularly important in Asia and Latin America, and is required to assess the actual extent of disease in these locations. Typhoid fever should be used as an example, when, despite the availability of vaccines, a lack of appropriate disease burden data stalled the global commitment, resulting in limited vaccine uptake and dampened efforts to develop conjugated vaccines. The persistence of typhoid fever culminated in the evolution of a highly antimicrobial resistant S. Typhi genotype (H58), which is spreading globally [55].
